# Exercise-Induced Bronchoconstriction in Non-Asthmatic Children with Moderate-to-Severe Persistent Allergic Rhinitis Undergoing Subcutaneous Allergen Immunotherapy: Prevalence and Risk Factors

**DOI:** 10.3390/jcm15155944

**Published:** 2026-07-30

**Authors:** Ercan Yılmaz, Nezihe Köker Özer, Erdem Topal

**Affiliations:** Department of Pediatric Allergy and Immunology, Faculty of Medicine, Inonu University, 44000 Malatya, Turkey; ercanyilmz44@gmail.com (E.Y.); erdemtopal44@gmail.com (E.T.)

**Keywords:** exercise-induced bronchoconstriction, subcutaneous allergen immunotherapy, allergic rhinitis, asthma

## Abstract

**Objective**: The aim of this study was to investigate the prevalence of exercise-induced bronchoconstriction and associated clinical risk factors in children in the early phase of subcutaneous allergen immunotherapy for moderate-to-severe persistent allergic rhinitis. **Methods**: This cross-sectional observational study included 62 children aged 6–18 years who were in the early phase of subcutaneous allergen immunotherapy for moderate-to-severe persistent allergic rhinitis. Patients with a diagnosis of asthma or asthma symptoms were excluded from the study. All participants underwent an exercise provocation test with spirometry in accordance with the recommendations of the American Thoracic Society and the European Respiratory Society. A decrease of ≥10% in FEV_1_ from baseline at any time point following exercise was defined as exercise-induced bronchoconstriction. Demographic characteristics, concomitant atopic diseases, laboratory parameters, nasal symptom scores and respiratory function tests were evaluated. **Results**: The median age of the patients was 14 (9–18) years, and 71% were male. Exercise-induced bronchoconstriction was detected in 21 (33.9%) patients following the exercise provocation test. Exercise-induced bronchoconstriction positivity was most frequently observed at the 15th minute post-exercise. A family history of asthma was significantly higher in the exercise-induced bronchoconstriction-positive group (23.8% versus 2.4%, *p* = 0.014). Furthermore, the baseline Visual Analogue Scale and Total Nasal Symptom Score were significantly higher in exercise-induced bronchoconstriction positive patients (*p* = 0.035 and *p* = 0.016, respectively). No significant differences were observed between the groups in terms of total IgE levels, peripheral eosinophil counts, multiple aeroallergen sensitization, and baseline spirometric parameters. **Conclusions**: The prevalence of latent exercise-induced bronchoconstriction was 33.9% among children with moderate-to-severe persistent allergic rhinitis without asthma symptoms who had initiated subcutaneous allergen immunotherapy. In particular, including an exercise provocation test in the assessment process for patients with a family history of asthma and a high nasal symptom score may contribute to the early diagnosis of subclinical lower airway involvement.

## 1. Introduction

Allergic rhinitis (AR) is one of the most common chronic allergic conditions in childhood and is characterized by IgE-mediated inflammation of the nasal mucosa. The condition can lead to significant consequences, including symptoms such as rhinorrhea, sneezing, nasal itching and congestion, as well as sleep disturbance, attention deficit, reduced academic performance and a decline in quality of life [1]. It is reported that the prevalence in childhood varies between 10 and 40% worldwide, and a marked increase has been observed in recent years, particularly in cases associated with pollen sensitivity [2].

It is now known that allergic rhinitis, which was previously considered an upper airway disease, is a systemic inflammatory process closely associated with the lower airways. The ‘one airway, one disease’ concept has demonstrated that the nasal and bronchial mucosa share similar histopathological characteristics and that inflammation in the upper airways can also affect the lower airways [3]. Consequently, bronchial hyperreactivity, small airway dysfunction and subclinical lower airway inflammation are frequently reported in individuals with allergic rhinitis [3,4].

The link between allergic rhinitis and asthma is well established. Whilst the vast majority of patients with asthma also have allergic rhinitis, it has been shown that children with allergic rhinitis have a significantly higher risk of developing asthma in later years compared to healthy children [4]. However, airway inflammation and bronchial hyperreactivity can also be detected in many children with allergic rhinitis who have not been clinically diagnosed with asthma. This supports the view that allergic rhinitis is not a disease limited solely to the upper airways [3,4].

Exercise-induced bronchoconstriction (EIB) is defined as a transient airway obstruction occurring following exercise [5]. Although EIB is frequently associated with asthma, it can also be observed in atopic individuals and, in particular, in children with allergic rhinitis [5,6]. The pathogenesis of exercise-induced bronchoconstriction is thought to involve multiple mechanisms, including airway water and heat loss secondary to exercise-induced hyperventilation, increased airway surface osmolarity, mast cell activation, and the release of inflammatory mediators [6]. Consequently, contraction occurs in the airway smooth muscles, leading to a bronchoconstrictive response.

It is known that the prevalence of EIB is higher in children with allergic rhinitis than in the general population. However, a significant proportion of these patients do not exhibit typical respiratory symptoms during exercise, and bronchial hyperreactivity may go unnoticed during standard clinical assessment [7]. This condition is described in the literature as ‘occult’ or ‘hidden’ EIB [7]. Failure to recognize hidden EIB may lead to a reduction in children’s physical activity capacity, a decline in quality of life, and the potential for early signs of asthma to be overlooked in later life.

In patients with moderate-to-severe persistent allergic rhinitis where symptom control is inadequate, subcutaneous allergen immunotherapy (SCIT) is recognized as the only causative treatment option capable of altering the natural course of the disease [8]. It has been demonstrated that SCIT can reduce symptoms, decrease medication use, and prevent the development of new sensitization [8,9]. Furthermore, some studies have reported that SCIT may reduce the risk of developing asthma [9,10]. For this reason, it is important to assess patients starting SCIT not only for upper airway symptoms but also for any potential associated lower airway involvement.

The detection of bronchial hyperreactivity is clinically significant, particularly in children with moderate-to-severe persistent allergic rhinitis who are candidates for SCIT. This is because subclinical lower airway involvement identified in this patient group may influence both treatment planning and long-term follow-up strategies. Nevertheless, the number of studies investigating the prevalence of latent EIB and associated risk factors in children starting SCIT is quite limited. The majority of existing studies include heterogeneous allergic rhinitis populations and have not focused on the high-risk patient group for whom immunotherapy is indicated.

Therefore, the aim of this study was to determine the prevalence of exercise-induced bronchoconstriction in children who have started subcutaneous allergen immunotherapy for moderate-to-severe persistent allergic rhinitis and to investigate the demographic, clinical and laboratory risk factors associated with the development of EIB.

## 2. Materials and Methods

### 2.1. Study Design and Patient Population

This cross-sectional observational study was conducted at a tertiary pediatric allergy and immunology center. The study included children and adolescents aged 6–18 years who had been diagnosed with moderate-to-severe persistent allergic rhinitis and were in the early phase of subcutaneous allergen immunotherapy (SCIT).

The diagnosis of allergic rhinitis was established based on characteristic clinical symptoms together with confirmed sensitization to aeroallergens, in accordance with the Allergic Rhinitis and its Impact on Asthma (ARIA) guidelines [2]. Moderate-to-severe persistent allergic rhinitis was defined by the presence of persistent symptoms associated with impairment in daily activities, sleep disturbance, and reduced school performance.

All participants had confirmed pollen sensitization and had commenced SCIT containing a grass pollen mixture. In all cases, immunotherapy was administered using the Roxall^®^ GrassMix preparation (ROXALL Medizin GmbH, Hamburg, Germany). The patients were followed at the pediatric allergy and immunology outpatient clinic, and pollen sensitization was confirmed based on clinical history, skin prick testing, and/or serum-specific IgE measurements.

Patients were screened for asthma symptoms, including recurrent wheezing, exertional breathlessness, night-time cough, occasional breathlessness, and a history of a previous asthma diagnosis by a doctor. Patients diagnosed with asthma, those reporting asthma symptoms, and those with chronic lung disease, congenital heart disease, primary immunodeficiency or systemic inflammatory disease, those who had suffered a respiratory tract infection within the previous four weeks, active smokers, patients with a baseline FEV_1_ value of less than 70% of the predicted value, and those who did not comply with or were unable to complete the study procedures were excluded from the study.

### 2.2. Clinical Assessment

Detailed demographic and clinical characteristics of all participants were recorded. Age, sex, family history, exposure to tobacco smoke, residence in a rural area, concomitant atopic diseases, family history of atopic diseases, history of breastfeeding, and pet ownership were assessed. Patients’ height and weight measurements were taken using standard methods, and height z-scores and weight z-scores were calculated according to age and gender. Anthropometric assessments were carried out in accordance with national reference data [11].

The severity of allergic rhinitis symptoms was assessed prior to subcutaneous allergen immunotherapy using the Visual Analogue Scale (VAS) and the Total Nasal Symptom Score (TNSS). In the VAS assessment, patients were asked to rate the overall severity of their symptoms on a scale of 0 to 10. When calculating the TNSS, each of the symptoms—nasal congestion, rhinorrhea, sneezing and nasal itching—was scored on a scale of 0–3, and a total score was obtained.

Laboratory data for all patients were obtained retrospectively from medical records. Peripheral blood eosinophil counts were recorded from complete blood count analyses, and total serum immunoglobulin E (IgE) levels were evaluated based on patients’ laboratory files and hospital records. Aeroallergen sensitivity was assessed using skin prick tests and/or serum-specific IgE measurements.

### 2.3. Respiratory Function Tests

Prior to the exercise provocation test, all participants underwent spirometry in accordance with the recommendations of the American Thoracic Society (ATS) and the European Respiratory Society (ERS) [12]. In the spirometric assessment, forced expiratory volume in the first second (FEV_1_), forced vital capacity (FVC), FEV_1_/FVC ratio, mid-expiratory flow rate (FEF_25–75_) and peak expiratory flow (PEF) values were recorded. At least three acceptable and reproducible maneuvers were obtained for each participant, and the highest values were used in the analyses. Results were expressed as percentages of the predicted values (% predicted) according to age, sex and height.

### 2.4. Exercise Provocation Test

The exercise provocation test was conducted in accordance with the guidelines on exercise-induced bronchoconstriction published by the American Thoracic Society [5]. All participants exercised on a treadmill, aiming to reach approximately 85–90% of their age-predicted maximum heart rate. The target exercise intensity was maintained for at least 6 min. Exercise testing was performed under standardized environmental conditions in an indoor laboratory, with an ambient temperature of 20–25 °C and relative humidity of <50%, in accordance with ATS recommendations [5]. To minimize potential pharmacological influences on airway responsiveness, participants were instructed to withhold medications before testing in accordance with ATS recommendations, including short-acting bronchodilators for at least 8 h, long-acting bronchodilators for at least 48 h, leukotriene receptor antagonists for at least 24 h, and other medications that could potentially influence airway responsiveness whenever clinically appropriate. Spirometric measurements were repeated at 5, 10, 15, and 30 min post-exercise. A decrease of 10% or more in FEV_1_ relative to the baseline value at any time point post-exercise was considered exercise-induced bronchoconstriction.

The change in FEV_1_ following exercise (ΔFEV_1_) was calculated as a percentage change relative to the baseline value. Symptoms such as dyspnea, cough, wheezing and chest tightness that arose during or after the test were recorded. Patients requiring bronchodilator treatment following the test were also recorded.

### 2.5. Sample Size

The primary endpoint of this study is to determine the prevalence of a positive exercise-induced bronchial provocation test in children with moderate-to-severe persistent allergic rhinitis who do not have asthma symptoms. Taking into account that the prevalence of exercise-induced bronchoconstriction in children with allergic rhinitis without a diagnosis of asthma has been reported in the literature to be approximately 20–30%, the expected prevalence was assumed to be 20%. In the sample size calculation for a single proportion, conducted at a 95% confidence level and with a 10% margin of error, the minimum sample size was calculated to be 62 patients.

### 2.6. Statistical Analysis

Statistical analysis was performed using SPSS Statistics (v.21.0; IBM Corp., Armonk, NY, USA). Normality was assessed using the Kolmogorov–Smirnov test. Data showing a normal distribution were presented as mean ± standard deviation, whilst data not showing a normal distribution were presented as median (minimum–maximum). For comparisons between groups, the independent samples *t*-test was used for variables with a normal distribution, and the Mann–Whitney U test was used for variables without a normal distribution. Categorical variables were compared using the Pearson’s chi-square test or Fisher’s exact test. Two-tailed *p*-values < 0.05 were considered statistically significant.

## 3. Results

### 3.1. Demographic and Clinical Characteristics of the Study Population

A total of 62 pediatric patients diagnosed with moderate-to-severe persistent allergic rhinitis and who had commenced subcutaneous grass mix allergen immunotherapy were included in the study. Forty-four (71%) of the patients were male, and the median age was 14 (9–18) years. The duration of allergic rhinitis symptoms was a median of 5 (2–10) years. The duration of allergen immunotherapy at the time of assessment was a median of 4 (1–8) months. A history of consanguineous marriage was present in 18 (29%) patients, passive smoking exposure in 11 (17.7%), and a history of living in a rural area in 24 (38.7%). Breastfeeding for more than six months was observed in 59 (95.2%) patients. A history of pet ownership was present in 6 (9.7%) patients. When assessing concomitant atopic conditions, allergic conjunctivitis was found to be the most common comorbidity (80.6%). A history of eczema was present in 6 patients (9.7%), a history of drug allergy in 2 (3.2%), and a history of food allergy in 1 (1.6%). Obesity was present in 7 (11.3%) patients. Upon examination of family history, a history of allergic rhinitis was found in 17 (27.4%) patients, asthma in 6 (9.7%) patients, and eczema in 2 (3.2%) patients. When the patients’ allergic rhinitis symptoms were examined, sneezing was the most common symptom, observed in 60 (96.8%) patients. This was followed by nasal itching in 59 (95.2%) patients, nasal discharge in 58 (93.5%) patients, and nasal congestion in 31 (50%) patients. When symptom severity prior to SCIT was assessed, the median baseline VAS score was 7 (3–10), and the baseline total nasal symptom score (TNSS) was 8 (3–12) (Table 1).

### 3.2. Laboratory Findings and Aeroallergen Sensitization Profile

In laboratory investigations, the median total IgE level was 314 (20–2000) IU/mL, whilst the median peripheral eosinophil count was 0.3 (0.1–3.7) × 10^9^/L. When the aeroallergen sensitization profile was assessed, pollen sensitization was detected in all 62 (100%) patients. In addition, 12 (19.3%) patients were sensitized to cat dander, 10 (16.1%) to house dust mites and 8 (12.9%) to mold. Multiple aeroallergen sensitization was detected in 12 (19.3%) patients (Table 1).

### 3.3. Pulmonary Function and Exercise Challenge Test Results

**Figure 1:**
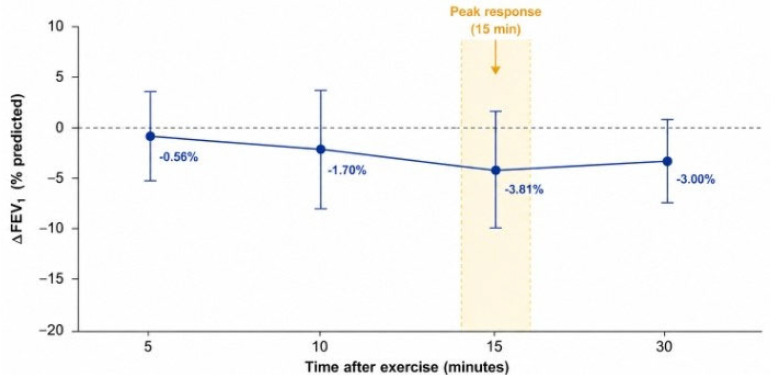
Mean change in FEV1 (% predicted) from baseline at 5, 10, 15, and 30 min after exercise challenge. ↓: Indicates the peak response (maximum mean decrease in FEV_1_) at 15 min after exercise.

In the baseline spirometric assessments performed prior to the exercise provocation test, the mean FEV_1_ value was 91.06% ± 8.08 of predicted, the FVC value was 95.77% ± 10.72 of predicted, and the FEV_1_/FVC ratio was 95.67% ± 8.55. The FEF_25–75_ value, indicative of small airway function, was 103.37% ± 20.59 of predicted, whilst the PEF value was 81.70% ± 14.26 of predicted. Overall, basal spirometric parameters were found to be within normal limits in the study group. As a result of the exercise provocation test, exercise-induced bronchoconstriction (EIB) was detected in 21 (33.9%) patients. When evaluated by time points, EIB positivity was most frequently observed at the 15 min mark. Accordingly, EIB positivity was detected in 3 (4.8%) patients at 5 min, 6 (9.7%) at 10 min, 15 (24.2%) at 15 min, and 8 (12.9%) at 30 min. When post-exercise changes in ΔFEV_1_ were examined, it was observed that the average decline was most pronounced at the 15 min mark (Figure 1). The mean ΔFEV_1_ change was calculated as 0.56 ± 3.71%, 1.70 ± 5.77%, 3.81 ± 5.46% and 3 ± 3.66% at 5, 10, 15 and 30 min, respectively. The most common symptom observed during the exercise provocation test was dyspnea, noted in 18 (29%) patients. Cough was noted in four (6.4%) patients, and wheezing in one (1.6%) patient. No patient reported a feeling of chest tightness. Following the test, only three (4.8%) patients required bronchodilator treatment (Table 2).

### 3.4. Risk Factors for Exercise-Induced Bronchoconstriction

When comparing EIB-positive and EIB-negative patients, no significant differences were found between the groups in terms of age, gender, duration of allergic rhinitis, concomitant allergic conjunctivitis, obesity, history of eczema, passive smoking exposure, pet ownership and living in a rural area (all *p* > 0.05). A family history of asthma was present in 5 (23.8%) patients in the EIB-positive group, whereas it was present in only 1 (2.4%) patient in the EIB-negative group, and this difference was statistically significant (*p* = 0.014, RR 1.28; 95% CI 1.003–1.634). Furthermore, baseline symptom scores were found to be higher in EIB-positive patients. The baseline VAS score was 7 (5–10) in the EIB-positive group and 6 (3–9) in the EIB-negative group (*p* = 0.035). Similarly, the baseline TNSS was 9 (6–12) in the EIB-positive group and 8 (3–12) in the EIB-negative group (*p* = 0.016). In terms of laboratory parameters, no significant differences were observed between the groups regarding total IgE levels, peripheral eosinophil count, and multiple aeroallergen sensitization (*p* = 0.112, *p* = 0.376, and *p* = 0.52, respectively). When baseline spirometric parameters were evaluated, there was no statistical difference between the two groups in terms of FEV_1_ and FEF_25–75_ values (*p* = 0.085 and *p* = 0.145, respectively) (Table 3).

## 4. Discussion

In this study, exercise-induced bronchoconstriction (EIB) was detected in 33.9% of children who had started subcutaneous allergen immunotherapy for moderate-to-severe persistent allergic rhinitis despite having no clinical symptoms of asthma. Furthermore, a family history of asthma, a higher baseline Visual Analogue Scale (VAS) score, and a higher baseline Total Nasal Symptom Score (TNSS) were significantly associated with EIB. In contrast, no significant associations were observed between EIB and age, sex, passive smoking exposure, total IgE level, peripheral eosinophil count, multiple aeroallergen sensitization, or baseline spirometric parameters. These findings indicate that a substantial proportion of children with moderate-to-severe persistent allergic rhinitis may have unrecognized EIB despite the absence of clinical asthma, highlighting the importance of considering objective exercise challenge testing in selected patients.

Although allergic rhinitis was long regarded as a condition affecting only the upper airways, it is now understood that both the upper and lower airways are affected by shared inflammatory mechanisms. This approach, defined as the ‘unified airway disease’ or ‘single airway, single disease’ model, demonstrates that the nasal and bronchial mucosa share similar immunological and histopathological characteristics [2,3,4]. It has been shown that the allergic inflammation occurring in the nasal mucosa is not merely a local process, but can also lead to eosinophilic inflammation, epithelial damage and airway hyperresponsiveness in the bronchial mucosa [2]. Consequently, impaired lower airway function may occur even in individuals clinically diagnosed with allergic rhinitis alone. The prevalence of EIB found in our study to be 33.9% supports this view. In previous studies conducted on children with allergic rhinitis, the prevalence of EIB has been reported to range between 15 and 45% [5,7,13]. However, in a significant proportion of these studies, the patient groups were heterogeneous and included cases with mild symptoms. In our study, however, all patients consisted of cases who had commenced immunotherapy due to moderate-to-severe persistent allergic rhinitis. This may have contributed to more pronounced allergic inflammation in the study group and, consequently, to the high prevalence of EIB.

A key finding of the present study was that 33.9% of children with moderate-to-severe persistent allergic rhinitis without asthma symptoms had exercise-induced bronchoconstriction. In daily clinical practice, these patients are often managed solely on the basis of a diagnosis of allergic rhinitis, and no assessment of the lower airways is carried out. However, our findings indicate that symptom assessment alone is insufficient to rule out bronchial hyperreactivity. This is particularly important in patients starting immunotherapy, as the subclinical bronchial hyperreactivity present in this patient group may serve as an early indicator of potential future asthma.

One of the notable findings of our study is that baseline VAS and TNSS scores were found to be significantly higher in EIB-positive patients. In other words, bronchial hyperreactivity was observed more frequently during exercise in patients with more severe nasal symptoms. This result suggests that there may be a direct relationship between upper airway inflammation and lower airway involvement. Previous studies have also reported that bronchial hyperreactivity is more common in patients with poor control of nasal symptoms [14,15]. In particular, it has been suggested that persistent nasal inflammation may increase the systemic inflammatory response, leading to inflammation in the bronchial mucosa. Our findings support this view.

Another significant finding in our study is that a family history of asthma was found to be strongly associated with EIB. It is well established that genetic predisposition plays a significant role in the development of asthma. Numerous studies have demonstrated that bronchial hyperreactivity, airway inflammation and atopic sensitization exhibit heritable characteristics [16,17]. Therefore, in children with moderate-to-severe persistent allergic rhinitis and a family history of asthma, the development of bronchial hyperreactivity can be expected even in the absence of clinically apparent symptoms. Our findings suggest that family history may serve as an important clinical indicator not only for the future development of asthma but also for identifying current subclinical lower airway involvement.

In our study, no significant association was found between total IgE levels, peripheral eosinophil counts, and multiple aeroallergen sensitization and EIB. Although this result may seem unexpected at first glance, there are studies in the literature reporting similar findings [18,19]. Although total IgE and peripheral eosinophil counts indicate systemic atopic inflammation, they may not always accurately reflect the degree of bronchial hyperreactivity. The local characteristics of airway inflammation, disruption of epithelial integrity and environmental factors may be more decisive in determining bronchial hyperreactivity. Therefore, it does not appear possible to predict lower airway involvement based solely on laboratory markers. One of the most clinically significant findings of our study is the basal spirometry results. No significant difference was observed between the EIB-positive and EIB-negative groups in terms of FEV_1_ and FEF_25–75_ values. Furthermore, in the vast majority of EIB-positive patients, baseline spirometric values were within normal limits. This finding demonstrates that normal spirometry results do not rule out bronchial hyperreactivity. Similarly, studies have reported that baseline spirometric parameters may be normal in many patients with a positive exercise provocation test [20,21,22,23]. Therefore, assessments based solely on resting spirometry may lead to the overlooking of clinically silent lower airway involvement.

In our study, the fact that the post-exercise decline in FEV_1_ was most pronounced at the 15 min mark is also consistent with the literature. It is known that post-exercise bronchoconstriction generally reaches its maximum level within the first 5–15 min [6]. The findings of our study support the view that the exercise protocol used was able to elicit a physiological bronchial hyperreactivity response. Furthermore, the fact that dyspnea is the most common symptom in EIB-positive patients suggests that some children may have airway narrowing that is not perceived during exercise but can be objectively demonstrated.

This study has several significant strengths. Firstly, the fact that it evaluated only children with moderate-to-severe persistent allergic rhinitis who had just started immunotherapy is one of the study’s unique features. The majority of studies in the literature include the general allergic rhinitis population and have not focused on the high-risk patient group eligible for immunotherapy. Furthermore, a standardized exercise provocation test was administered to all patients, and a wide range of clinical, laboratory and functional variables were assessed together.

The present study has several limitations. First, its single-center, cross-sectional design and relatively small sample size limit the generalizability of the findings and preclude establishing temporal or causal relationships between allergic rhinitis and exercise-induced bronchoconstriction (EIB). Accordingly, our results should be interpreted as demonstrating an association rather than causality. Second, EIB was defined using a ≥10% decline in FEV_1_ after exercise in accordance with ATS recommendations. Although this threshold is widely accepted, different diagnostic cut-offs proposed in the literature may yield different prevalence estimates. Third, bronchial hyperresponsiveness was assessed solely by exercise provocation testing, and no comparative evaluation with other bronchial challenge tests, such as methacholine or mannitol, was performed. In addition, biomarkers reflecting airway inflammation, such as fractional exhaled nitric oxide (FeNO), were not evaluated. Finally, because of the cross-sectional design, we were unable to determine whether allergen immunotherapy modifies the prevalence or severity of EIB over time or whether EIB-positive children subsequently develop clinical asthma. Future prospective longitudinal studies with larger cohorts are warranted to clarify the long-term effects of allergen immunotherapy on exercise-induced airway narrowing, lower airway inflammation, and subsequent respiratory outcomes.

## 5. Conclusions

In conclusion, exercise-induced bronchoconstriction was present in 33.9% of children and adolescents with moderate-to-severe persistent allergic rhinitis during the early phase of subcutaneous allergen immunotherapy, despite the absence of clinically diagnosed asthma. These findings support an association between allergic rhinitis and subclinical lower airway involvement. Exercise challenge testing may represent a useful tool for identifying children at increased risk of lower airway dysfunction; however, prospective longitudinal studies are needed to confirm its clinical utility and to determine whether allergen immunotherapy is associated with changes in exercise-induced bronchoconstriction over time.

## Figures and Tables

**Table 1:** Demographic, clinical and laboratory characteristics of the children initiating subcutaneous allergen immunotherapy for moderate-to-severe persistent allergic rhinitis (*n* = 62).

Variable	*n* (%)
Male sex	44 (71)
Age, years, median (min–max), years	14 (9–18)
Symptom duration, median (min–max), years	5 (2–10)
Duration of allergen immunotherapy, median (min–max), months	4 (1–8)
Consanguineous marriage	18 (29)
Passive smoking exposure	11 (17.7)
Breast feed >6 month	59 (95.2)
Pet in house	6 (9.7)
Living in a rural area	24 (38.7)
Co-morbid allergic conjunctivitis	50 (80.6)
Eczema history	6 (9.7)
Drug allergy history	2 (3.2)
Food allergy history	1 (1.6)
Co-morbid obesity	7 (11.3)
Asthma	6 (9.7)
Allergic rhinitis	17 (27.4)
Eczema	2 (3.2)
Itching	59 (95.2)
Sneezing	60 (96.8)
Discharge	58 (93.5)
Congestion	31 (50)
Baseline VAS score before SCIT, median (min–max)	7 (3–10)
Baseline TNSS before SCIT, median (min–max)	8 (3–12)
Total Ig E, median (min–max), IU/mL	314 (20–2000)
Eosinophil count, median (min–max) × 10^9^/L	0.3 (0.1–3.7)
Pollen	62 (100)
House dust mite	10 (16.1)
Mold	8 (12.9)
Cat dander	12 (19.3)
Multiple aeroallergen sensitization	12 (19.3)

SCIT: Subcutaneous allergen immunotherapy, TNSS: Total Nasal Symptom Score, VAS: Visual Analogue Scale.

**Table 2:** Exercise challenge test findings in children initiating subcutaneous allergen immunotherapy for moderate-to-severe persistent allergic rhinitis.

Variables	*n* (%)
Basal spirometry	
FEV_1_ (% predicted), mean ± SD	91.06 ± 8.08
FVC (% predicted), mean ± SD	95.77 ± 10.72
FEV_1_/FVC (%), mean ± SD	95.67 ± 8.55
FEF_25–75_ (% predicted), mean ± SD	103.37 ± 20.59
PEF (% predicted), mean ± SD	81.70 ± 14.26
Exercise challenge test results	
EIB positivity at 5 min, *n* (%)	3 (4.8)
EIB positivity at 10 min, *n* (%)	6 (9.7)
EIB positivity at 15 min, *n* (%)	15 (24.2)
EIB positivity at 30 min, *n* (%)	8 (12.9)
Overall, EIB positivity, *n* (%)	21 (33.9)
Post-exercise ΔFEV_1_ (%) at each assessment time point	
ΔFEV_1_ (%) predicted, mean ± SD, at 5 min	0.56 ± 3.71
ΔFEV_1_ (%) predicted, mean ± SD, at 10 min	1.70 ± 5.77
ΔFEV_1_ (%) predicted, mean ± SD, at 15 min	3.81 ± 5.46
ΔFEV_1_ (%) predicted, mean ± SD, at 30 min	3 ± 3.66
Exercise-related symptoms during testing	
Cough	4 (6.4)
Wheezing	1 (1.6)
Dyspnea	18 (29)
Chest tightness	-
Bronchodilator administration after test, *n* (%)	3 (4.8)

EIB: Exercise-induced bronchoconstriction, FEV_1_: Forced expiratory volume in the first second, FVC: Forced vital capacity, FEF_25–75_: Mid-expiratory flow rate, PEF: Peak expiratory flow.

**Table 3:** Comparison of Children with and without Exercise-Induced Bronchoconstriction.

Variables	EIB-Positive Group, (*n* = 21)	EIB-Negative Group, (*n* = 41)	*p* Value
Age	13 (12–17)	14 (9–18)	0.717
Male sex, *n* (%)	13 (61.9)	31 (75.6)	0.407
Duration of allergic rhinitis	5 (2–10)	5 (3–10)	0.454
Co-morbid allergic conjunctivitis	15 (71.4)	35 (85.4)	0.308
Co-morbid obesity	3 (14.3)	4 (9.8)	0.68
History of eczema	2 (9.5)	4 (9.8)	1.0
History of food allergy	-	1 (2.4)	NC
History of drug allergy	-	2 (4.9)	NC
Family history of asthma	5 (23.8)	1 (2.4)	0.014
Family history of allergic rhinitis	6 (28.6)	11 (26.8)	0.884
Family history of eczema	-	2 (4.9)	NC
Breast feed >6 month	19 (90.5)	40 (97.6)	0.263
Passive smoking exposure	6 (28.6)	5 (12.2)	0.160
Pet in house	2 (9.5)	4 (9.8)	1.0
Living in a rural area	11 (52.4)	13 (31.7)	0.191
Baseline VAS score before SCIT, median (min–max)	7 (5–10)	6 (3–9)	0.035
Baseline TNSS score before SCIT, median (min–max)	9 (6–12)	8 (3–12)	0.016
Total IgE, median (min–max), IU	314 (20–1646)	328 (180–2000)	0.112
Eosinophil count, median (min–max) × 10^9^/L	0.18 (0.1–3.7)	0.37 (0.1–1.19)	0.376
Multiple aeroallergen sensitization, *n* (%)	5 (23.8)	7 (17)	0.52
Baseline FEV_1_, (% predicted), mean ± SD	94.09 ± 9.87	89.51 ± 6.60	0.085
Baseline FEF_25–75_, (% predicted), mean ± SD	97.57 ± 20.65	106.34 ± 20.16	0.145

SCIT: Subcutaneous allergen immunotherapy, EIB: Exercise-induced bronchoconstriction, NC: Not calculated.

## Data Availability

The data presented in this study are available on request from the corresponding author.
